# High Prevalence of West Nile Virus in Domestic Birds and Detection in 2 New Mosquito Species in Madagascar

**DOI:** 10.1371/journal.pone.0147589

**Published:** 2016-01-25

**Authors:** Marianne Maquart, Sébastien Boyer, Vincent Michel Rakotoharinome, Julie Ravaomanana, Michael Luciano Tantely, Jean-Michel Heraud, Eric Cardinale

**Affiliations:** 1 Unité de Virologie, Institut Pasteur de Madagascar, Antananarivo, Madagascar; 2 Unité d’Entomologie Médicale, Institut Pasteur de Madagascar, Antananarivo, Madagascar; 3 Direction des Services Vétérinaires, Ministère de l’Elevage, Antananarivo, Madagascar; 4 Centre National de la Recherche Appliquée au Développement Rural, -Département de Recherches Zootechniques et Vétérinaires, Antananarivo, Madagascar; 5 Centre de coopération Internationale en Recherche Agronomique pour le Développement, UMR 15 CMAEE, Sainte Clotilde, La Réunion, France; 6 Institut National de la Recherche Agronomique, UMR 1309 CMAEE, Sainte Clotilde, La Réunion, France; 7 Centre de Recherche et de Veille sur les maladies émergentes dans l’Océan Indien, Sainte Clotilde, La Réunion, France; University of California Davis, UNITED STATES

## Abstract

West Nile virus is an arthropod-borne zoonosis transmitted by a large number of mosquito species, and birds play a key role as reservoir of the virus. Its distribution is largely widespread over Africa, Asia, the Americas and Europe. Since 1978, it has frequently been reported in Madagascar. Studies described a high seroprevalence level of the virus in humans in different areas of the island and a human fatal case of WNV infection was reported in 2011. Despite these reports, the epidemiology of WNV in Madagascar, in particular, viral circulation remains unclear. To explore the transmission of WNV in two rural human populations of Madagascar, we investigated local mosquitoes and poultry for evidence of current infections, and determined seroprevalence of candidate sentinel species among the local poultry. These 2 areas are close to lakes where domestic birds, migratory wild birds and humans coexist. Serological analysis revealed WNV antibodies in domestic birds (duck, chicken, goose, turkey and guinea fowl) sampled in both districts (Antsalova 29.4% and Mitsinjo 16.7%). West Nile virus nucleic acid was detected in one chicken and in 8 pools of mosquitoes including 2 mosquito species (*Aedeomyia madagascarica* and *Anopheles pauliani*) that have not been previously described as candidate vectors for WNV. Molecular analysis of WNV isolates showed that all viruses detected were part of the lineage 2 that is mainly distributed in Africa, and were most closely matched by the previous Malagasy strains isolated in 1988. Our study showed that WNV circulates in Madagascar amongst domestic birds and mosquitoes, and highlights the utility of poultry as a surveillance tool to detect WNV transmission in a peri-domestic setting.

## Introduction

West Nile virus (WNV) is a zoonotic arbovirus affecting humans, horses and wildlife. The virus is widely distributed in Africa, Asia and Europe and spread over the last 2 decades to North and South America [1]. WNV is a flavivirus described as 8 lineages but only lineages 1 and 2 are involved in important human outbreaks [2]. Lineage 1 includes viruses from Saharan Africa, Europe, Asia, Australia and North, Central and South America while lineage 2 was mainly detected in Africa and Madagascar [3, 4]. Although in humans, WNV associated disease is more often characterized by a febrile stage with myalgia, arthralgia and lymphadenopathy, a less common manifestation is neurological disease, which can be fatal [5, 6]. WNV is maintained within a bird-mosquito-bird transmission cycle. Some bird species, like American crow (*Corvus brachyrhynchos*) and house sparrow (*Passer domesticus*) develop a sufficient viremia titer to transmit the virus to mosquitoes, while others like the domestic rock pigeon (*Columba livia*) and the barn owl (*Tyto alba*) do not [7, 8]. These birds are considered as amplifiers with a key role in the epidemiology of the virus and can as act reservoirs. Domestic birds like chickens do not develop sufficient viremia to permit a transmission cycle and so are considered as dead end hosts. Poultry species have been used as surveillance sentinels in many geographic regions [9] but their role of WNV transmission and surveillance value has not been well-investigated at this time.

In Madagascar, WNV lineage 2 was first isolated in 1978 from an endemic parrot species (*Coracopsis vasa*) [10]. To date, ten mosquito species are considered to be the main WNV vectors in Madagascar. The last inventory of WNV amongst Malagasy mosquitoes performed from 1978 to 1988 showed that 12 species were infected with WNV including 2 *Anopheles* species, 4 *Aedes* species and 6 *Culex* species [11]. The possible involvement of *Anopheles* species is quite surprising. However, to date, 23 *Anopheles* species are already described in Madagascar with a highly zoophilic behavior, including on birds, and could occasionally feed on humans (unpublished data).

In 1990, a serosurvey study conducted amongst people aged from 5 to 20 years-old in Madagascar showed that 29.9% of sera tested positive for WNV antibodies [12]. A second serosurvey conducted in 1996 on children younger than 15 years-old in the highlands, and in 1999 in the north-western coast showed that 2.1% and 10.6% tested positive, respectively [13]. These data suggested the virus has been circulating for years in various areas of Madagascar. More recently, in 2011 a woman returning from Madagascar died on Reunion Island from WNV neuroinvasive disease [6]. Subsequently, our study’s purpose is to evaluate WNV exposure in candidate sentinel species among local poultry species by determining seroprevalence, a measure of past infection, and investigating local mosquitoes as its candidate vectors, thus exploring its potential circulation in two human populations in rural Madagascar.

## Materials and Methods

### Study sites

Two study areas were selected in two Western regions of Madagascar: Mitsinjo and Antsalova districts. These districts correspond to ecotypes in which human population, domestic and wild migratory wild birds coexist, and the presence of potential mosquito vectors and WNV circulation has been previously reported (i.e., positive serological results and viral detection).

In Mitsinjo district, the Kinkony Lake represents the second biggest lake of Madagascar and is a stopover site for migratory birds and is also home to resident waterfowl. Three villages were investigated in November and December 2012: Marofandroboka, Ankisaosy and Mahakary close to the Kinkony Lake

In Antsalova district, three major lakes composed a national park where wild birds are present year-round. Four villages, belonging to the Masoarivo municipality, were investigated in July 2013: Antsakoramby and Ankirangato close to Soamalipo lake, and Masoarivo and Mananga around the Antsamaka Lake.

### Domestic birds sampling

Serum samples (400μl) were collected by venipuncture in the wing vein on all domestic healthy birds brought by villagers (i.e., chicken (*Gallus gallus domesticus*), duck (*Cairina moschata*), turkey (*Meleagris gallopavo*), goose (*Anser anser domesticus*), and guinea fowl (*Numida meleagris*). All these animals were resident in the villages without travel history outside the village. Birds are considered as juvenile when the farmer reported it as younger than 5 months old. All serum samples were centrifuged in the field, transferred in liquid nitrogen to the laboratory, and stored at -80 C until use.

### Ethics statement

Bird trapping, handling, and sampling were implemented with the approval of national veterinary authorities and did not involve endangered or protected species. Farmers in each zone gave a verbal consent to participate into our study and gave permission for the blood sample collection from birds on their property. Our study protocol and procedures were approved by the committee of the Livestock ministry of Madagascar which is the sole relevant authority for animal care in Madagascar. The ethical committee number is: 2012/WN/Minel/3. We followed the European guidelines (European directives EU 86/609-STE123 and 2010/63/EU) for animal handling. Sampling was exclusively done by veterinarians and the animals were blood-sampled without suffering and were subsequently released. No animals were sacrificed during the study.

### Entomological survey

During the same period of time in which domestic birds were sampled and within the same study sites, mosquitoes were collected for testing. Mosquito traps were placed near the lake shore and in the villages. Mosquitoes were captured in CDC light traps (BioQuip Products, Inc., Rancho Dominguez, USA), net traps baited with chickens, BG-Sentinel baited with chickens (Biogents, Regensburg, Germany) [14], human landing, or a backpack aspirator (BioQuip Products, Inc., Rancho Dominguez, USA), and identified and stored as previously described [15]. In Mitsinjo district, four villages were investigated: Marofandroboka, Amboanjo, Morafeno and Mahakary. In Antsalova district, two villages were investigated (Masoarivo and Antsakoramby) as well as in the vicinity of the Antsamaka lake.

### Serological analysis

Bird serum samples were tested using IDScreen West Nile Competition Multi-species ELISA (enzyme‐linked immunosorbent assay) (IdVet, France) according to manufacturer's guidance, to detect immunoglobulins M and G (IgM and IgG) [16].

### Virological analysis

Viral detection was carried out on pools containing 10 sera from 10 different individual birds. Viral RNA was extracted from 140 μl of a pool of 10 sera (14 μl of each serum), by using the QIAamp Viral RNA Mini Kit (Qiagen, Hilden, Germany), according to the manufacturer’s protocol. Positive pools were further analysed individually with the aim of identifying individual positive sera.

Molecular analysis was performed on mosquito pools that contained 1 to 34 monospecific unfed female mosquitoes homogenized in 750 μl of cell culture medium (MEM) (containing 40% fetal bovine serum, 2 mM l-glutamine, 1000 U/mL penicillin, 100 mg/mL streptomycin, and 2.5 mg/mL amphotericin B). Grinding was performed by shaking the pools twice at 25 Hz frequency for 2 min with a 5-mm stainless steel ball (Dejay Distribution Ltd., Crowborough, United Kingdom) in TissueLyser II (Qiagen, Crawley, United Kingdom). Viral RNA was then extracted from 140 μl of the supernatants using the QIAamp Viral RNA Mini Kit (Qiagen, Hilden, Germany). The 60 μl eluted RNA was stored at -80°C.

The molecular detection of the virus was performed on a T3000 Biometra thermocycler using a semi-nested RT-PCR. The first round of PCR used primers WN132 and WN240 described by Berthet et al. [17] giving a 328 bp product and the second round used the forward primer WN132 and the in-house degenerated primer WN ESN: 5’- CTCCAKGGSAGGTTSAGRTCCAT—3’ giving a 228 bp product. Briefly, RT-PCR and the first round of PCR were performed in a one-step process on 4 μl of resuspended RNA template using the SuperScript III One-step RT-PCR reaction mix (Invitrogen). The second round of PCR was performed on 1μL of primary PCR product in a final volume of 25 μL containing 2X GoTaq^®^ Hot start Mastermix (Promega, Madison, USA) and 500nM of primers WN 132 and WN ESN. Cycling conditions of the second round of PCR were as follows: 95°C*#x2013;5min, then 25 cycles of 95°C–45 sec / 53°C–45 sec / 72°C–45 sec, followed by a final elongation step at 72°C–**12 min. When a sample tested positive, a second extraction and RT-PCR from the original sample was processed to confirm the result.**

### Sequence and phylogenetic analysis

Positive amplicons were purified on a 1% (w/v) agarose gel by electrophoresis with the QIAquick gel extraction kit (Qiagen), and sequenced commercially (Macrogen, Korea).

For the determination of WNV genotype of the Malagasy strains, a total of 42 WNV GenBank partial sequences of 228 bp of the envelope gene and 1 partial sequence of JEV were selected according to their origin (Asia, Europe, Africa and America), their host (human, birds, mosquitoes) and their lineage. Basic Local Alignment Search Tool (BLAST) alignments were generated using MUSCLE [18] through the Mega 5 software [19]. A phylogenetic tree was generated by neighbour-joining distance analysis with node values generated by 1000 bootstraps replications, using the Mega 5 software [19].

### Statistical analysis

Data were recorded and analyzed statistically with the R software package (version 3.0.1) [20]. The importance of main factors (sex, species, age (adult or juvenile) of animals, district and commune of origin) to the outcome of animals seroprevalence were evaluated with a regression analysis with multiple variables.

## Results

### Field collection

**Fig 1:**
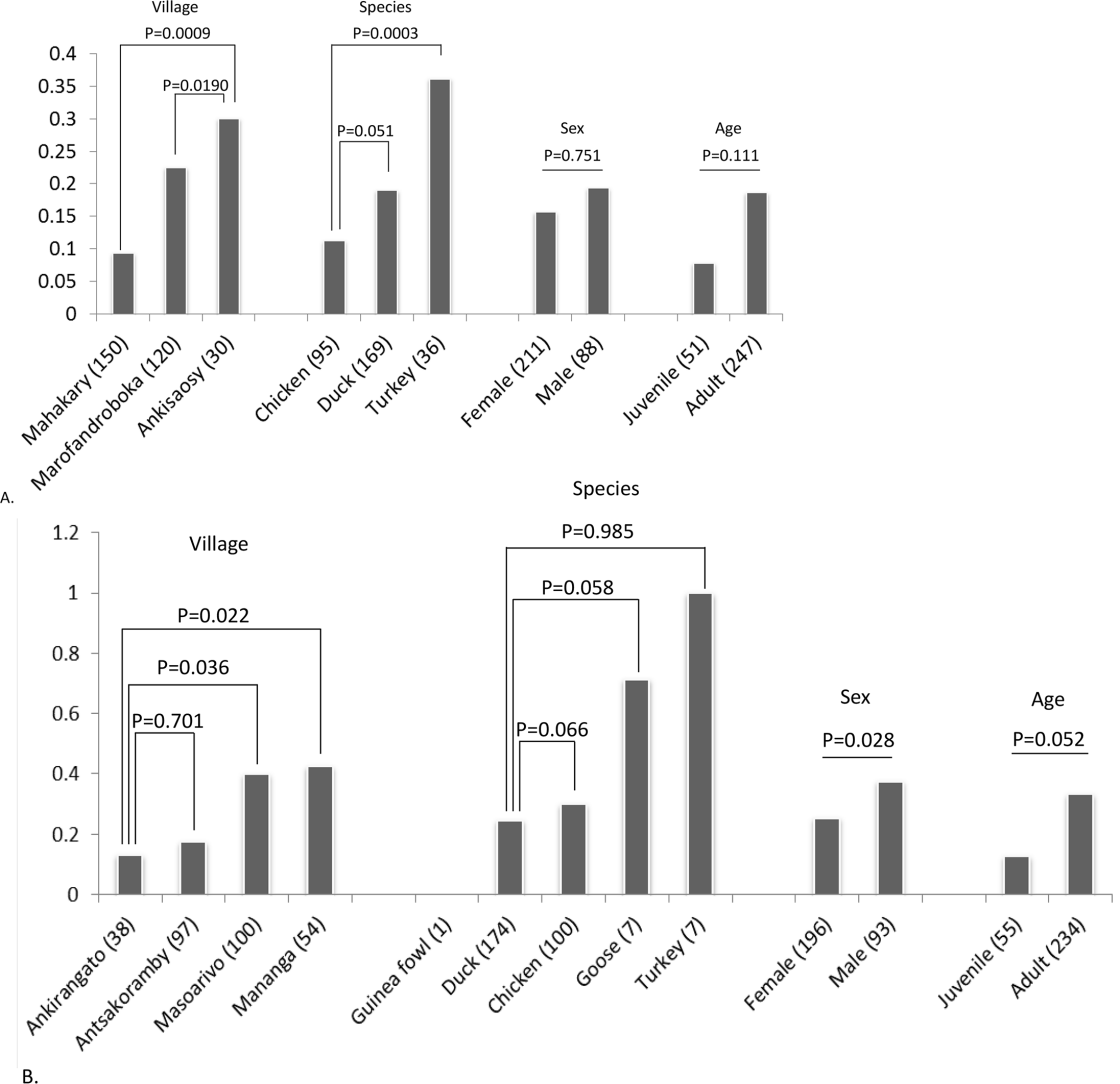
Seroprevalence analysis of West Nile virus in domestic birds. (A) in the Mitsinjo district (B) in the Antsalova district. Numbers in parentheses corresponded to the number of animals in each category and in the y axis represented the percentage of animals presenting WNV antibodies. P-values were generated from the regression analysis.

A total of 589 animals were sampled and bled, 300 in Mitsinjo and 289 in Antsalova districts respectively. In Mitsinjo district, most of the birds were sampled in Mahakary (n = 150) and Marofandroboka (n = 120) villages. In Antsalova district, animals were mainly sampled in Masoarivo (n = 100) and Antsakoramby (n = 97) villages. Ducks (n = 343), chickens (n = 195) and turkeys (n = 43) comprised the majority of birds sampled in both districts. In Antsalova district, geese and guinea fowls were also sampled. Sampling information is presented in Fig 1. The mosquito sampling results are briefly described below and a study on the efficacy of the various techniques utilized is presented elsewhere [15].

In Mitsinjo, 453 adult mosquitoes from 20 species (including 10 potential WNV vectors [15]) were collected during 7 consecutive nights (Table 1). In Masoarivo municipality, 1,014 adult mosquitoes from 19 species (including 10 potential WNV vectors) were caught during three consecutive nights (Table 1).

**Table 1:** Number of adult female mosquitoes, by species, caught in the Mitsinjo district and the Antsalova district.

Species	Antsalova	Mitsinjo
Aedeomyia madagascarica*	4	12
***Aedes aegypti***	0	8
***Aedes albocephalus***	0	109
*Aedes albodorsalis*	0	3
*Aedes durbanensis*	0	8
*Aedes fowleri*	0	8
*Aedes moucheti*	18	0
***Anopheles coustani***	4	2
*Anopheles funestus*	59	9
*Anopheles fuscicolor*	103	0
*Anopheles gambiae sl*	1	17
*Anopheles grassei*	0	11
***Anopheles maculipalpis***	1	0
Anopheles pauliani*	9	2
*Anopheles pharoensis*	4	94
*Anopheles squamosus*	0	1
*Culex annulioris*	0	1
***Culex antennatus***	98	11
*Culex bitaeniorhynchus*	0	14
***Culex decens***	2	0
***Culex pipiens***	0	10
***Culex poicilipes***	268	0
***Culex tritaeniorhynchus***	20	62
***Culex univitattus***	1	56
*Ficalbia circumtestacea*	9	0
*Mansonia africana*	1	0
***Mansonia uniformis***	**407**	**15**
*Uranotaenia alboabdominalis*	1	0
*Uranotaenia balfouri*	4	0
Total	598	439

Species represented in bold are candidate vectors of WNV and species indicated by an asterisk represented potential new candidate vectors identified in the study.

### Serological analysis

Differences in seroprevalence rates were observed among villages, as well as differences in species and age (Fig 1). In Mitsinjo district, WNV seroprevalence in birds in Mahakary village was significantly lower than the prevalence in Maforandroboka and Ankisaosy villages (Fig 1A). In the Antsalova district, Antsakoramby and Ankirangato villages showed lower seroprevalence than in Masoarivo and Mananga villages (Fig 1B).

Overall, in both areas, turkeys presented the higher seroprevalence rate than other species sampled. No statistical difference was observed between ducks and chickens. In the Antsalova district, geese sampled also presented a high seroprevalence rate but the number of animals tested was low (n = 7). Only one guinea fowl was assessed and tested negative.

In the two study sites, seroprevalence rates increased with age of animals despite non-significance. A slightly higher seroprevalence is observed in males than in females in Antsalova district.

### Molecular analysis

One of 60 pools of avian sera tested positive for WNV RNA; this pool contained sera from adult chickens from Marofondroboka, part of the Mitsinjo district. Eight of 121 mosquito pools tested positive for WNV nucleic acid.

In the Antsalova district, one pool of *Anopheles pauliani* and four pools of *Mansonia uniformis* were WNV-positive. In the Mitsinjo district, three pools of mosquitoes were WNV-positive: one pool of *Aedeomyia madagascarica* and two pools of *Anopheles coustani*.

### Phylogenetic analysis

**Fig 2:**
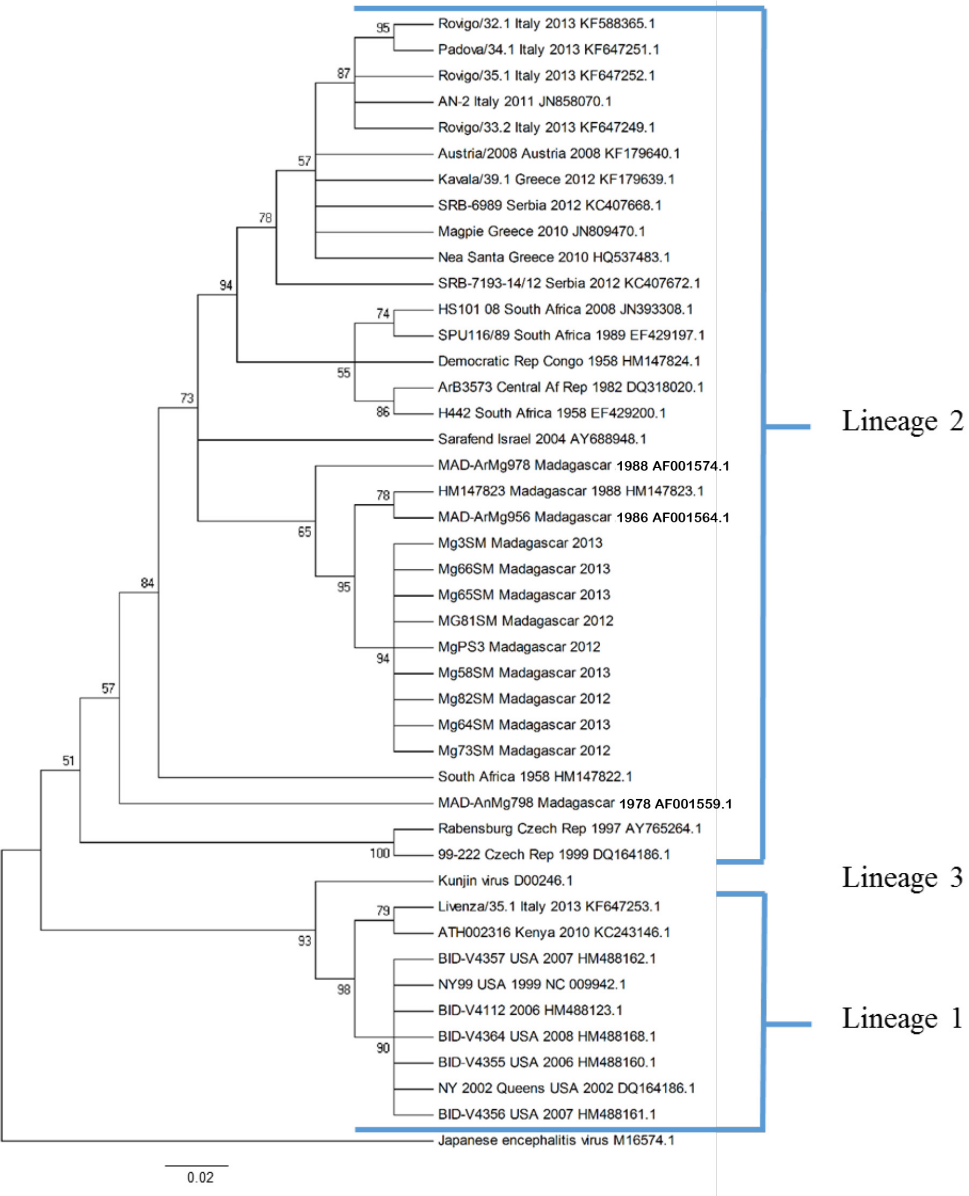
Genetic relatedness of geographically distinct WNV isolates determined by using the nucleotide sequence data from a 228 bp region of the E gene. The tree was constructed with the PAUP by using the neighbor-joining distance program of Mega 5 software. Node values were determined for 1,000 replicates. Isolates are labeled as follows: strain identification, country, date of isolation, genbank accession number.

A partial sequence of 228 bp was obtained for the 9 Malagasy isolates (8 from mosquito pools and 1 from a chicken serum). The 9 sequences are deposited in the Genbank database under the accession numbers: KP099553, KP099554, KP099555, KP099556, KP099557, KP099558, KP099559, KP099560 and KP099561. The topology showed two distinct lineages. The 9 Malagasy isolates belonged to lineage 2 (Fig 2). They also branched with the strains already detected in Madagascar (99% nucleotide homology), forming a distinct Malagasy clade (including strains isolated for the first time in 1978), with a high node support value (posterior probability pp = 0.95).

## Discussion

Our results confirm domestic poultry are exposed to WNV in Madagascar and describe two new mosquito species as potential vector for WNV. The diversity of species trapped is described by Boyer et al. (2014) and many of these species (especially C*ulex* and *Anopheles* species) were previously described as WNV potential vectors [15]. The two study sites were characterized by different abundances and species composition of mosquitoes. Differences in the abundance and the diversity of wildlife around villages could be one of the numerous factors influencing the variation in WNV seroprevalence of poultry in Antsalova and Mitsinjo. Molecular analysis of vector pools indicated current circulation of the virus in mosquitoes in the west region of Madagascar. Viral RNA was identified in 4 mosquito species (*Anopheles coustani*, *Anopheles pauliani*, *Mansonia uniformis*, and *Aedeomyia madagascarica*). *Anopheles coustani* and *M*. *uniformis* have been detected with WNV in Israel and Ethiopia, respectively [21]; however, the present study describes the first detection of WNV in these species in Madagascar. Interestingly, *Aedeomyia madagascarica* and *Anopheles pauliani*, 2 endemic species have not been previously documented with WNV infection. Furthermore, the 4 mosquito species *An*. *coustani*, *An*. *pauliani*, *Ma*. *uniformis*, and *Aed*. *madagascarica* are already known as zoo-anthropophagous insects, increasing the possibility of WNV interaction among human, mosquito vectors, and other potential WNV amplifying hosts in these particular ecotypes. *Aed*. *Madagascarica* and *An*. *pauliani* are known to preferentially feed on avian blood while *Ma*. *Uniformis* and *An*. *coustani* generally prefer cattle in Madagascar.

Cross reactivity can be observed within flaviviruses family. However, the ELISA kit used in this study presented a good sensitivity and specificity [16]. Furthermore, excellent agreement was demonstrated between serum neutralization (SN) test (considered as the reference test) and this ELISA kit, further supporting the validity of ELISA results and the conclusions drawn for them [22]. A high seroprevalence was observed with geese and turkeys and it was significantly different from chicken and ducks, although the low number of specimens sampled limited the interpretation. Chicken and turkeys are not good amplifier hosts, with low viremia, not really good for mosquito contamination [23]. Turkeys were more likely to seroconvert than other poultry species in the village ecosystem, although this may be due to their older age compared to chickens, and therefore longer exposure to infected mosquitoes [12]. Consequently, turkeys and young chickens may be the best choices as sentinel birds to detect a current circulation. Moreover, viral RNA was detected in a chicken serum. This rare event has already been evidenced by Petrovic et al. [24].

We detected most of WNV positive mosquito pools from villages located close to the lakes where lakeshores with emergent vegetation may support mosquito breeding. However, we also found that some villages like Masoarivo, Mananga and Marofondroboka (that are not located at the lake edge) exhibited high prevalence rates in poultry, despite lower numbers of trapped mosquitoes in these regions during the same time frame. Different hypotheses could be proposed to explain such a situation; firstly, the villages are not so far from the lakes and temporary waterholes favorable to mosquitoes. Secondly, the timing of WNV infections of birds cannot be determined; birds over a year of age may have been infected in years prior to the study [12]. Thirdly, wild birds are found everywhere, and many are adapted to humanized ecosystems and mosquitoes have the ability to move from a site to another, increasing the risk of infection throughout these relatively small areas.

Phylogenetic analysis revealed that all isolates detected in our study belonged to lineage 2, in accordance with the global repartition of the different WNV lineages [3, 4]. In Madagascar, all isolates achieved since 1978 have been closely related, suggesting local circulation of WNV presumably maintained by wild birds, acting as a reservoir of the virus, and transmitted by various potential mosquito vectors of the virus.

Finally, this study highlighted local exposure of WNV in domestic birds in the villages. Most of all, with 2 new identified WNV candidate vectors, the number of mosquito species that serve as potential vectors of the virus increases to 28 in Madagascar (Tantely, personal communication). A study is consequently in progress in the Mitsinjo district to assess the role of wild birds and mosquitoes in the virus transmission, identify a possible seasonality of WNV and evaluate the risk for humans.
